# The effect of age on ventilation management and clinical outcomes in critically ill COVID–19 patients––insights from the PRoVENT–COVID study

**DOI:** 10.18632/aging.203863

**Published:** 2022-01-31

**Authors:** Liselotte Hol, Paula Van Oosten, Sunny Nijbroek, Anissa Tsonas, Michela Botta, Ary Serpa Neto, Frederique Paulus, Marcus Schultz, PRoVENT–COVID, Collaborative group

**Affiliations:** 1 Department of Anesthesiology, Amsterdam University Medical Centers, University of Amsterdam, Amsterdam, Netherlands; 2 Department of Intensive Care, Amsterdam University Medical Centers, University of Amsterdam, Amsterdam, Netherlands; 3 Department of Critical Care Medicine, Hospital Israelita Albert Einstein, São Paolo, Brazil; 4 Department of Critical Care Medicine, Austin Hospital and University of Melbourne, Melbourne, Australia; 5 Faculty of Health, ACHIEVE, Center of Applied Research, University of Applied Research, Amsterdam, Netherlands; 6 Mahidol Oxford Tropical Medicine Research Unit (MORU), Mahidol University, Bangkok, Thailand; 7 Nuffield Department of Medicine, University of Oxford, Oxford, United Kingdom

**Keywords:** age, coronavirus disease 2019, COVID-19, critical care, invasive ventilation, mortality

## Abstract

Introduction: We analyzed the association of age with ventilation practice and outcomes in critically ill COVID–19 patients requiring invasive ventilation.

Methods: Posthoc analysis of the PRoVENT–COVID study, an observational study performed in 22 ICUs in the first 3 months of the national outbreak in the Netherlands. The coprimary endpoint was a set of ventilator parameters, including tidal volume normalized for predicted bodyweight, positive end–expiratory pressure, driving pressure, and respiratory system compliance in the first 4 days of invasive ventilation. Secondary endpoints were other ventilation parameters, the use of rescue therapies, pulmonary and extrapulmonary complications in the first 28 days in the ICU, hospital– and ICU stay, and mortality.

Results: 1122 patients were divided into four groups based on age quartiles. No meaningful differences were found in ventilation parameters and in the use of rescue therapies for refractory hypoxemia in the first 4 days of invasive ventilation. Older patients received more often a tracheostomy, developed more frequently acute kidney injury and myocardial infarction, stayed longer in hospital and ICU, and had a higher mortality.

Conclusions: In this cohort of invasively ventilated critically ill COVID–19 patients, age had no effect on ventilator management. Higher age was associated with more complications, longer length of stay in ICU and hospital and a higher mortality.

## INTRODUCTION

The coronavirus disease 2019 (COVID–19) pandemic has resulted in worldwide recurrent surges of patients in need for urgent and intense medical care [1], and as of early–November 2021 5 million patients have died from this new disease [2]. Many hospitalized COVID–19 patients need admission to an intensive care unit (ICU), most often for escalation of respiratory support that includes invasive ventilation [3].

Aging is associated with various changes in lung physiology [4]. Due to changes in the structure of the thoracic cage, aging is known to reduce chest wall compliance. However, lung compliance increases with age because of a decrease in elastic recoil. Second, aging is associated with so–called ‘senile emphysema’ [5]. Due to a decrease in the supporting structures of lung parenchyma, the risk for early closure of small airways increases which could result in air trapping. The increased incidence of comorbidities in elderly may also mandate a different ventilation approach. For example, the combination of a reduced respiratory system reserve and an increased incidence of pulmonary disease in elderly patients may require a higher FiO_2_, while the higher incidence of cardiovascular disease in the elderly may actually reduce the possibility of, for example, ventilation with higher pressures. Indeed, one small prospective cohort study showed that elderly patients with acute respiratory failure received ventilation with lower pressures compared to younger patients [6]. However, this was not confirmed by a more recently published study, showing no age dependent variations in ventilator settings in such patients [7].

Several risk factors for contracting severe COVID–19 have been identified and described. Elderly patients, but also patients with underlying cardiovascular or respiratory conditions are most vulnerable to develop a complicated SARS–CoV–2 infection [8–10], and are at a higher risk for mortality of this disease [11–13]. Aging itself, however, is linked to the development of comorbidities and functional disabilities. Indeed, patients aged > 65 years are three times more often diagnosed with multiple chronic diseases [14], including comorbidities like cancer, cardiovascular diseases, and diabetes mellitus. All these are well–known predictors for mortality [15–17]. Older age is also associated with immunological alterations and inflammation, which may also translate into a higher risk of dying from an infectious disease [16].

It is unknown whether age–related differences exist in ventilator settings in critically ill COVID–19 patients. It also remains uncertain to which extent the association of age with mortality in COVID–19 patients requiring invasive ventilation is mediated by the increased prevalence of comorbidities in elderly patients. In the context of these uncertainties, we assessed the database of a large national observational study [18, 19]. We hypothesized that age has an independent effect on ventilator management and has an association with outcome in critically ill invasively ventilated COVID–19 patients.

## MATERIALS AND METHODS

### Design, study sites, and participants

This is a posthoc analysis of a national multicenter observational study, named ‘Practice of VENTilation in COVID–19 patients’ (PRoVENT–COVID) [18]. This study included more than 40% of all critically ill COVID–19 patients admitted to a Dutch ICU in the first 3 months of the national outbreak. The study protocol was approved by the Institutional Review Board of the Amsterdam UMC, location AMC, Amsterdam, the Netherlands on 7 April 2020 (W20_157 # 20.171), and hence at the other 21 hospital that eventually participated in the study. The need for written informed consent was waived because of the observational nature. The study was registered at clinicaltrials.gov (study identifier NCT04346342).

Adult patients were eligible if admitted to the ICU of a participating hospital, and receiving invasive ventilation for respiratory failure related to COVID–19, confirmed by RT–PCR. For the current analysis, we excluded patients that were transferred to an ICU in a non–participating hospital within the first hour of invasive ventilation.

### Data collection

Multiple in–person and virtual meetings were organized at the Amsterdam University Medical Centers, location ‘AMC’, to train data collectors, that were all doctors in training or medical residents. During these meetings, data entry instructions were given, the database structure was explained, and data entry was trained. Each data collector was supervised by an experienced researcher in the domain of critical care. If inaccuracies, outliers and errors were found after data review, queries were sent and resolved by local investigators. Patient characteristics, anthropometric data, medical history, and available severity scores as recorded in the electronic patient records, severity of acute respiratory distress syndrome (ARDS) according to the current Berlin definition for this syndrome [20], and the extent of lung involvement on chest computed tomography or chest radiographs was collected for all patients at baseline. Different disease severity scores, e.g., the Acute Physiology and Chronic Health Evaluation (APACHE) II or IV score, the Simplified Acute Physiology Score (SAPS) II and the Sequential Organ Failure Assessment (SOFA) score, were used in the participating hospitals. The disease severity score documented in each hospital was collected at baseline, i.e., in the first 24 hours in the ICU. Laboratory test results, hemodynamic parameters, kidney function, fluid balance, and use and dose of continuous sedation, muscle paralysis, and vasopressors were captured daily up to calendar day 4.

Ventilator settings and key ventilation variables and parameters, and the use of adjunctive rescue therapies for refractory hypoxemia, including alveolar recruitment maneuvers, prone positioning, use of neuromuscular blocking agents (NMBAs), and extracorporeal membrane oxygenation (ECMO) was collected at fixed time points 3 times per day (08:00, 16:00 and 24:00) up to calendar day 4 or until death or ICU discharge, if that occurred first. From these three measurement points, the daily mean was calculated for each respiratory variable.

Pulmonary and extrapulmonary events were recorded until ICU day 28, ICU discharge or date of death, whichever came first.

Patients’ location and life status were collected up to day 90.

### Study endpoints

The coprimary endpoint of this current analysis was a set of 4 key ventilator settings and ventilation parameters: tidal volume normalized for predicted bodyweight (V_T PBW_), positive end–expiratory pressure (PEEP), driving pressure (ΔP), and respiratory system compliance (Crs) during the first 4 calendar days.

Secondary endpoints were other ventilation parameters and use of rescue therapies for hypoxemia, pulmonary and extrapulmonary complications, ICU and hospital discharge, the number of days alive and free from invasive ventilation at day 28, and mortality at ICU and hospital discharge and at day 28 and 90.

### Definitions

Pulmonary and extrapulmonary events were defined as pneumothorax, tracheostomy, reintubation, acute kidney injury and need for renal replacement therapy, and thromboembolic events, including pulmonary embolism, deep venous thrombosis, ischemic stroke, myocardial infarction, and systemic arterial thrombosis.

V_T_ per predicted bodyweight (PBW) was calculated as follows:

(females) PBW (kg)=45.5+0.91∗(height [cm]−152.4) [eq. 1a];

(males) PWB (kg)=50.0+0.91∗(height [cm]−152.4) [eq. 1b]; and

$${\text{V}}_{\text{T, PBW}}\;(\text{ml/kg})={\text{V}}_{\text{T}}\;(\text{ml})\text{/PBW}\;(\text{kg})$$ [eq. 2].

ΔP and mechanical power (MP) were calculated using the following equations:

$$\begin{array}{l} \text{ΔP}\;(\text{cm}\;{\text{H}}_{2}\text{O})=\text{peak}\;\text{pressure}\;(\text{Ppeak})\\ \left(\text{cm}\;{\text{H}}_{2}\text{O})-\text{PEEP}\;(\text{cm}\;{\text{H}}_{2}\text{O}\right) \end{array}$$ [eq. 3]; and

$$\begin{array}{l} \text{MP}\;(\text{J/min})=0.098*{\text{V}}_{\text{T}}(\text{liters})*\text{respiratory}\;\text{rate}\;(\text{RR})\\ {}*(\text{Ppeak}-0.5*\Delta P) \end{array}$$ [eq. 4]

Crs was calculated as follows:

$$\text{Crs}\;(\text{ml/cm}\;{\text{H}}_{2}\text{O})={\text{V}}_{\text{T}}\left(\text{ml}\right)/\Delta P\;(\text{cm}\;{\text{H}}_{2}\text{O})$$ [eq. 5]

### Power calculation

We did not perform a formal power calculation––instead, the number of patients available in the database was used as the sample size.

### Statistical analysis plan

Patients were categorized into 4 age groups using the age quartiles. The day of the start of ventilation was merged with the first full calendar and named ‘day 1’. The following days were named ‘day 2’ and ‘day 3’. No assumptions for missing data were made.

Categorical variables are presented as numbers and proportions, continuous variables are reported with median and interquartile ranges. Age groups were compared using the Kruskal–Wallis test for continuous variables and Fisher exact tests for categorical variables. If differences were found, a posthoc Dunn test was used for pairwise comparison.

Distribution plots were constructed to show the key ventilator parameters for the four age groups. Time-to-event outcomes are presented in Kaplan–Meier curves, and age groups are compared with the Log–rank test.

To adjust for the unequal distribution of effect modifiers between the 4 age groups, multivariable models were made for ICU and hospital mortality, and 28– and 90-day mortality. The following variables were considered for adjustment in these models: (i.) gender; (ii.) body mass index (BMI); (iii.) history of hypertension, heart failure, diabetes mellitus, chronic kidney disease, chronic obstructive pulmonary disease, active hematological or solid cancer; (iv.) use of angiotensin-converting enzyme inhibitors, use of angiotensin II receptor blockers, and use of vasopressor or inotropic medication; (v.) PaO_2_ to FiO_2_ ratio; and (vi.) mean arterial blood pressure, heart rate, plasma creatinine, fluid balance, and arterial pH. These baseline covariates were selected according to clinical relevance and as used in previous studies [18, 21].

All analyses were conducted in R, version 4.0.5. A *P* < 0.05 was considered statistically significant.

## RESULTS

### Participants

Patient flow is shown in Supplementary Figure 1. A total of 1340 patients in 22 ICUs were screened for eligibility; major reasons for exclusions were that patients had an alternate diagnosis or did not receive invasive ventilation. Of the remaining 1122 patients, the median age was 65 [57 to 72] years. Baseline demographics of the 4 age groups are presented in Table 1. Older patients were shorter, weighed less, had a lower BMI and were more often diagnosed with a medical history of arterial hypertension, heart failure, diabetes mellitus, or COPD. Home medication like angiotensin–converting enzyme inhibitors and blockers, beta–blockers, statins, and calcium channel blockers were more often used at home in the higher age groups. At the first day of invasive ventilation, older patients were more often in need of vasopressors and inotropic drugs, and older patients had a higher cumulative fluid balance and a lower urine output.

**Table 1:** Patient characteristics according to age category at baseline.

	**Age 22 to 57 years *(n =* 287)**	**Age 58 to 65 years *(n =* 286)**	**Age 66 to 72 years *(n =* 283)**	**Age 73 to 85 years *(n =* 266)**	***P* value**
Age, years	52.0 [47.0 to 55.0]	62.0 [60.0 to 64.0]	69.0 [67.0 to 71.0]	75.0 [74.0 to 77.0]	<0.001
Male	200 (69.7)	217 (75.9)	203 (71.7)	197 (74.1)	0.370
Height, cm	178.0 [170.0 to 185.0]	178 [170.0 to 184.0]	175.0 [170.0 to 180.0]	174.0 [168.5 to 180.0]	<0.001
Weight, kg	90.0 [80.8 to 105.0]	89.0 [78.2 to 98.0]	85.0 [75.6 to 92.2]	82.0 [75.0 to 90.0]	<0.001
Body Mass Index, kg/m^2^	28.9 [26.2 to 32.7]	27.7 [25.4 to 30.6]	27.2 [24.8 to 29.7]	27.0 [24.9 to 29.4]	<0.001
Severity of illness*					
SAPS II, % (no)	35.7 (99/277)	34.3 (92/268)	33.6 (91/271)	30.8 (77/250)	
*Modified SAPS II	24.0 [19.0 to 29.0]	24.0 [19.0 to 31.0]	24.5 [19.0 to 32.0]	26.0 [20.0 to 34.0]	0.361
APACHE II, no (%)	26.0 (72/277)	25.4 (68/268)	17.7 (48/271)	22.4 (56/250)	
*Modified APACHE II	12.0 [10.0 to 15.0]	12.0 [9.0 to 15.0]	15.0 [9.0 to 19.0]	15.0 [10.0 to 20.0]	0.026
APACHE IV, no (%)	45.5 (126/277)	40.7 (109/268)	41.7 (113/271)	36.8 (92/250)	
*Modified APACHE IV	44.0 [37.2 to 55.0]	44.0 [35.0 to 56.5]	49.0 [36.8 to 59.2]	49.0 [34.8 to 62.0]	0.469
SOFA, no (%)	53.4 (148/227)	54.1 (145/268)	46.5 (126/271)	44.4 (111/250)	
SOFA	7.0 [5.0 to 8.0]	7.0 [6.0 to 10.0]	7.0 [6.0 to 10.0]	8.0 [7.0 to 12.5]	<0.001
Comorbidities					
Arterial hypertension	53 (18.5)	105 (36.7)	108 (38.2)	114 (42.9)	<0.001
Heart failure	3 (1.0)	10 (3.5)	16 (5.7)	20 (7.5)	<0.001
Diabetes mellitus	44 (15.3)	62 (21.7)	80 (28.3)	64 (24.1)	0.002
Chronic kidney disease	8 (2.8)	14 (4.9)	9 (3.2)	16 (6.0)	0.204
Baseline creatinine	71.0 [60.0 to 87.0]	77.0 [64.0 to 98.0]	78.0 [63.0 to 98.0]	84.0 [66.8 to 111.2]	<0.001
Liver cirrhosis	2 (0.7)	0 (0.0)	0 (0.0)	1 (0.4)	0.329
Chronic obstructive pulmonary disease	8 (2.8)	25 (8.7)	34 (12.0)	21 (7.9)	<0.001
Active hematological neoplasia	3 (1.0)	5 (1.7)	4 (1.4)	4 (1.5)	0.911
Active solid neoplasia	3 (1.0)	7 (2.4)	8 (2.8)	10 (3.8)	0.193
Neuromuscular disease	4 (1.4)	0 (0.0)	2 (0.7)	2 (0.8)	0.258
Immunosuppression	7 (2.4)	8 (2.8)	5 (1.8)	4 (1.5)	0.710
Previous medication					
Systemic steroids	6 (2.1)	8 (2.8)	10 (3.5)	14 (5.3)	0.216
Inhalation steroids	34 (11.8)	37 (12.9)	33 (11.7)	21 (7.9)	0.244
Angiotensin-converting enzyme inhibitor	25 (8.7)	45 (15.7)	62 (21.9)	57 (21.4)	<0.001
Angiotensin II receptor blocker	18 (6.3)	35 (12.2)	30 (10.6)	44 (16.5)	0.002
Beta-blockers	28 (9.8)	52 (18.2)	63 (22.3)	68 (25.6)	<0.001
Insulin	16 (5.6)	22 (7.7)	21 (7.4)	19 (7.1)	0.744
Metformin	29 (10.1)	47 (16.4)	52 (18.4)	47 (17.7)	0.020
Statins	35 (12.2)	76 (26.6)	110 (38.9)	109 (41.0)	<0.001
Calcium channel blockers	29 (10.1)	45 (15.7)	59 (20.8)	64 (24.1)	<0.001
Transferred under invasive ventilation from another hospital	59 (20.6)	53 (18.5)	48 (17.0)	41 (15.4)	0.436
Days between admission and start of invasive ventilation	0.0 [0.0 to 0.0]	0.0 [0.0 to 0.0]	0.0 [0.0 to 0.0]	0.0 [0.0 to 0.0]	0.508
Use of non-invasive mechanical ventilation before intubation	28/259 (10.8)	14/256 (5.5)	24/258 (9.3)	19/236 (8.1)	0.152
Duration of non-invasive ventilation, hours	7.0 [2.0 to 23.0]	7.0 [3.5 to 19.0]	8.0 [2.8 to 9.5]	8.0 [1.0 to 17.0]	1.000
Chest CT-scan performed at baseline	111/276 (40.2)	93/270 (34.4)	78/269 (29.0)	81/257 (31.5)	0.023
Percentage lung parenchyma affected					0.561
0%	7/111 (6.3)	3/93 (3.2)	3/78 (3.8)	1/81 (1.2)	
25%	29/111 (26.1)	27/93 (29.0)	29/78 (37.2)	31/81 (38.3)	
50%	38/111 (34.2)	26/93 (28.0)	21/78 (26.9)	22/81 (27.2)	
75%	30/111 (27.0)	33/93 (35.5)	19/78 (24.4)	22/81 (27.2)	
100%	7/111 (6.3)	4/93 (4.3)	6/78 (7.7)	5/81 (6.2)	
Chest x-ray performed at baseline	136/162 (84.0)	152/176 (86.4)	157/185 (84.9)	157/176 (89.2)	0.506
Quadrants affected					0.810
1	13 (9.8)	12 (7.8)	8 (5.0)	9 (5.8)	
2	32 (24.1)	37 (24.0)	38 (23.8)	32 (20.8)	
3	34 (25.6)	39 (25.3)	45 (28.1)	50 (32.5)	
4	54 (40.6)	66 (42.9)	69 (43.1)	63 (40.9)	
Laboratory tests					
pH	7.4 [7.3 to 7.4]	7.4 [7.3 to 7.4]	7.4 [7.3 to 7.4]	7.3 [7.3 to 7.4]	<0.001
PaO_2_	10.7 [9.2 to 14.2]	10.3 [8.8 to 12.6]	10.9 [9.5 to 13.3]	11.2 [9.7 to 13.3]	0.008
SaO_2_	95.0 [93.0 to 97.4]	94.2 [92.0 to 96.8]	95.0 [93.0 to 97.0]	95.0 [93.0 to 97.0]	0.030
PaCO_2_	5.6 [4.9 to 6.5]	5.9 [5.0 to 6.9]	6.1 [5.3 to 7.1]	5.9 [5.0 to 6.9]	0.003
Lactate	1.1 [0.9 to 1.4]	1.1 [0.9 to 1.4]	1.2 [0.9 to 1.5]	1.2 [1.0 to 1.6]	0.002
Worst PaO_2/_FiO_2_ ratio, mm Hg	126.6 [94.7 to 164.5]	117.9 [91.8 to 160.3]	120.2 [96.1 to 157.3]	126.2 [97.4 to 161.6]	0.401
Need for advanced support					
Continuous sedation	277/287 (96.5)	276/286 (96.5)	267/277 (95.0)	253/263 (95.1)	0.691
Need for vasopressor use	198/287 (69.0)	223/286 (78.0)	225/281 (80.1)	217/266 (81.6)	0.002
Need for inotropic use	6/287 (2.1)	6/286 (2.1)	16/281 (5.7)	17/266 (6.4)	0.009
Fluid balance, mL	418.0 [-126.0 to 1206.0]	513.0 [-26.3 to 1209.0]	456.1 [-25.5 to 1252.8]	780.0 [144.0 to 1557.0]	0.001
Urine output, mL	875.0 [511.2 to 1377.5]	657.0 [350.0 to 1120.0]	720.0 [370.0 to 1165.0]	505.0 [255.0 to 877.5]	<0.001

Data presented as median with interquartile range [25^th^ to 75^th^ quartile] or n (%). *Age component is removed from the APACHE and SAPS Score. *Total numbers are different because different scores were used in the participating hospitals.

SAPS, Simplified Acute Physiology Score; APACHE, Acute Physiology and Chronic Health Evaluation; SOFA, Sequential Organ Failure Assessment; CT, Computed Tomography.

### Ventilation characteristics

**Figure 1:**
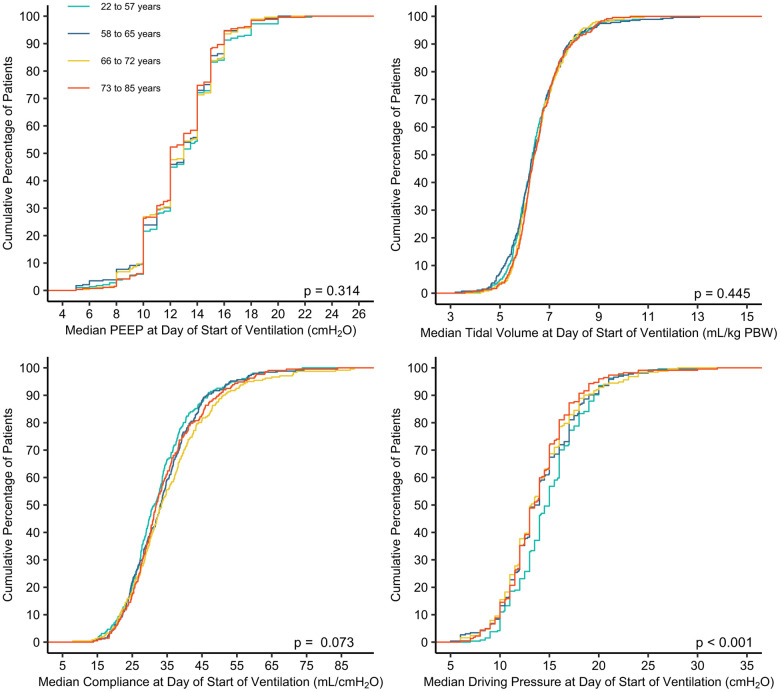
**Cumulative frequency distribution of median PEEP, tidal volume, compliance and driving pressure at start day of invasive ventilation.** Mean values were calculated from three or four measurements available on the first day of ventilation. The Kruskal-Wallis test was used to calculate p-values.

Key ventilator settings are shown in Table 2, Figure 1, and Supplementary Figures 2–5. On the first day of ventilation, median V_T PBW_, PEEP, ΔP and Crs were largely similar between the 4 age groups. Some differences reached statistical significance, but differences were too small to have a clinical meaning.

**Table 2:** Characteristics of mechanical ventilation and laboratory results in the first day of ventilation.

	**Age 22 to 57 years *(n =* 287)**	**Age 58 to 65 years *(n =* 286)**	**Age 66 to 72 years *(n =* 283)**	**Age 73 to 85 years *(n =* 266)**	***P* value**
Mode of mechanical ventilation					
Volume control	32/271 (11.8)	35/267 (13.1)	33/267 (12.4)	41/248 (16.5)	0.398
Pressure control	163/271 (60.1)	153/267 (57.3)	149/267 (55.8)	123/248 (49.6)	0.103
Pressure support	12/271 (4.4)	20/267 (7.5)	13/267 (4.9)	12/248 (4.8)	0.380
Synchronized Intermitted Mandatory Ventilation	19/271 (7.0)	12/267 (4.5)	25/267 (9.4)	22/248 (8.9)	0.131
Airway Pressure Release Ventilation	9/271 (3.3)	10/267 (3.7)	10/267 (3.7)	5/248 (2.0)	0.652
INTELLIVENT-Adaptive Support Ventilation	11/271 (4.1)	10/267 (3.7)	12/267 (4.5)	11/248 (4.4)	0.971
Other	25/271 (9.2)	27/267 (10.1)	25/267 (9.4)	34/248 (13.7)	0.310
Use of assisted ventilation	76/287 (26.5)	78/282 (27.7)	88/283 (31.1)	88/265 (33.2)	0.285
Tidal volume (n/N), mL/kg PBW*	(274/287) 6.4 [5.8 to 7.0]	(274/286) 6.4 [5.9 to 7.1]	(263/283) 6.5 [5.9 to 7.1]	(243/266) 6.5 [6.0 to 7.1]	0.445
PEEP, (n/N) cmH2O*	(287/287) 13.0 [11.0 to 15.0]	(286/286) 12.7 [11.0 to 14.6]	(279/283) 13.0 [10.7 to 14.8]	(262/266) 12.2 [10.8 to 14.2]	0.314
Driving pressure (n/N), cmH2O*	(264/287) 14.7 [12.5 to 17.0]	(265/286) 13.8 [11.7 to 16.3]	(252/283) 13.2 [11.3 to 15.7]	(227/266) 13.5 [11.6 to 15.7]	<0.001
Compliance (n/N), mL/cmH2O*	(256/287) 32.4 [25.9 to 38.3]	(258/286) 33.8 [27.1 to 41.7]	(241/283) 34.7 [27.7 to 43.3]	(215/266) 32.6 [27.3 to 40.7]	0.073
Mechanical power (n/N), J/min*	(256/287) 19.2 [16.0 to 23.7]	(257/286) 19.3 [15.9 to 23.1]	(241/283) 17.9 [14.7 to 22.3]	(214/266) 17.2 [14.6 to 20.9]	<0.001
Peak pressure (n/N), cmH_2_O*	(264/287) 27.7 [25.0 to 30.8]	(267/286) 26.7 [23.3 to 30.0]	(257/283) 26.0 [23.3 to 29.2]	(227/266) 26.2 [23.6 to 29.0]	<0.001
Total respiratory rate (n/N), breaths per minute*	(287/287) 22.0 [20.0 to 24.3]	(286/286) 22.0 [19.5 to 24.5]	(282/283) 21.3 [19.3 to 24.0]	(258/266) 21.3 [19.1 to 23.7]	0.053
Minute ventilation (n/N), L/min*	(275/287) 9.8 [8.6 to 11.4]	(277/286) 10.0 [8.5 to 11.6]	(269/283) 9.6 [8.2 to 11.3]	(245/266) 9.3 [8.2 to 10.6]	0.005
Minute volume corrected (n/N), mL/kg/min PBW*	(274/287) 139.1 [121.9 to 158.3]	(274/286) 139.9 [124.8 to 162.9]	(263/283) 137.7 [123.7 to 159.6]	(243/266) 137.2 [122.8 to 155.0]	0.782
FiO_2_ (n/N)*	(286/287) 0.6 [0.5 to 0.7]	(286/286) 0.6 [0.5 to 0.7]	(281/283) 0.6 [0.5 to 0.7]	(258/266) 0.6 [0.5 to 0.7]	0.283
PaO_2_ (n/N), mmHg*	(284/287) 81.0 [71.5 to 99.3]	(286/286) 78.7 [71.3 to 93.4]	(280/283) 82.4 [72.7 to 95.4]	(264/266) 83.3 [75.0 to 96.0]	0.018
PaCO_2_ (n/N), mmHg*	(284/287) 42.9 [38.3 to 48.4]	(286/286) 44.6 [39.8 to 49.5]	(280/283) 46.1 [39.9 to 52.0]	(264/266) 45.0 [39.1 to 50.9]	0.002
EtCO_2_ (n/N), mmHg*	(264/287) 38.0 [33.8 to 43.8]	(257/286) 37.7 [33.3 to 42.8]	(261/283) 36.3 [31.9 to 42.0]	(231/266) 35.3 [31.6 to 39.9]	<0.001

Data presented as median with interquartile range [25^th^ to 75^th^ quartile] or n (%). *Mean of all values available at the first day of ventilation. Total numbers are different because of missing or unmeasured values. EtCO_2_, End-Tidal Carbon Dioxide; FiO_2_, inspired fraction of oxygen; ICU, Intensive Care.

Mechanical power and peak pressure decreased from the younger to the older age groups at the first day of ventilation (Table 2). The difference in mechanical power and peak pressure disappeared in subsequent days (Supplementary Table 1). EtCO_2_ was lower but PaCO_2_ was higher in older age groups, and PaO_2_ was lower in the second age quartile (Table 2); only the difference in EtCO_2_ persisted in subsequent days (Supplementary Table 1) Use of adjunctive therapies for refractory hypoxemia was not affected by age, except for the use of NMBAs, which was less used with higher age (Table 3).

**Table 3:** Clinical outcome according to age group.

	**Age 22 to 57 years *(n =* 287)**	**Age 58 to 65 years *(n =* 286)**	**Age 66 to 72 years *(n =* 283)**	**Age 73 to 85 years *(n =* 266)**	***P* value**
28-day mortality	36/281 (12.8)	59/279 (21.1)	100/279 (35.8)	123/263 (46.8)	<0.001
90-day mortality	46/255 (18.0)	72/251 (28.7)	120/267 (44.9)	145/242 (59.9)	<0.001
In hospital mortality	43/259 (16.6)	71/256 (27.7)	113/255 (44.3)	140/252 (55.6)	<0.001
ICU mortality	42/277 (15.2)	71/278 (25.5)	110/274 (40.1)	133/262 (50.8)	<0.001
Length of hospital stay, days	24.0 [17.0 to 33.0]	26.0 [16.0 to 41.0]	22.0 [14.0 to 39.0]	21.5 [10.0 to 36.0]	0.008
Length of hospital stay in survivors, days	25.0 [18.5 to 35.5]	30.0 [20.0 to 46.5]	32.5 [20.3 to 49.8]	33.0 [25.8 to 52.0]	<0.001
Length of ICU stay, days	15.0 [10.0 to 23.0]	17.0 [10.0 to 30.0]	16.0 [8.3 to 26.0]	14.0 [7.0 to 25.0]	0.037
Length of ICU stay in survivors, days	15.0 [10.0 to 22.8]	20.0 [12.0 to 31.0]	18.0 [10.0 to 34.0]	20.0 [13.0 to 38.0]	<0.001
Ventilator-free days at day 28	13.0 [0.0 to 19.0]	4.0 [0.0 to 17.0]	0.0 [0.0 to 14.2]	0.0 [0.0 to 9.7]	<0.001
Duration of ventilation, days	13.0 [9.0 to 21.0]	15.0 [9.0 to 26.0]	15.0 [8.0 to 24.0]	13.0 [6.0 to 22.0]	0.023
Duration of ventilation in survivors, days*	13.0 [8.0 to 21.2]	17.0 [10.0 to 28.3]	17.0 [10.0 to 31.0]	19.0 [12.0 to 34.0]	<0.001
Tracheostomy*	35/283 (12.4)	62/284 (21.8)	48/280 (17.1)	45/265 (17.0)	0.029
Reintubation*	32/282 (11.3)	42/284 (14.8)	33/278 (11.9)	33/264 (12.5)	0.631
Pneumothorax*	2/283 (0.7)	3/275 (1.1)	2/267 (0.7)	2/259 (0.8)	0.970
Thrombotic complications*^&^	72/287 (25.1)	95/286 (33.2)	74/283 (26.1)	78/266 (29.3)	0.135
Pulmonary embolism	55/287 (19.2)	75/286 (26.2)	61/283 (21.6)	58/266 (21.8)	0.236
Deep vein thrombosis	17/287 (5.9)	20/286 (7.0)	9/283 (3.2)	11/266 (4.1)	0.156
Ischemic stroke	3/287 (1.0)	10/286 (3.5)	8/283 (2.8)	10/266 (3.8)	0.148
Myocardial infarction	2/287 (0.7)	0/286 (0.0)	7/283 (2.5)	7/266 (2.6)	0.007
Systemic arterial thrombosis	1/287 (0.3)	1/286 (0.3)	2/283 (0.7)	0/266 (0.0)	0.805
Acute kidney injury*	89/287 (31.0)	140/285 (49.1)	126/281 (44.8)	141/265 (53.2)	<0.001
Need for renal replacement*	35/287 (12.2)	62/286 (21.7)	57/283 (20.1)	51/266 (19.2)	0.013
Adjunctive therapies refractory hypoxemia**	162/284 (57.0)	174/282 (61.7)	159/282 (56.4)	152/265 (57.4)	0.563
Prone positioning	156/284 (54.9)	169/282 (59.9)	155/282 (55.0)	145/265 (54.7)	0.533
Alveolar recruitment maneuver	15/242 (6.2)	16/239 (6.7)	18/239 (7.5)	15/214 (7.0)	0.946
Other adjunctive therapies**	156/287 (54.4)	134/286 (46.9)	143/283 (50.5)	104/266 (39.1)	0.003
Neuromuscular blocking agents	156/287 (54.4)	133/286 (46.5)	141/283 (49.8)	104/266 (39.1)	0.003
Extracorporeal membrane oxygenation	7/285 (2.5)	2/282 (0.7)	2/278 (0.7)	1/262 (0.4)	0.142

Data presented as median with interquartile range [25^th^ to 75^th^ quartile] or n (%). Totals are different due to missing data. *Assessed at day 28. **Assessed in the first four days of ventilation. ^&^One could have more than one thrombotic complication. Total numbers are different because of missing or unmeasured values.

ICU, Intensive Care Unit.

### Pulmonary and extrapulmonary events

Pulmonary and extrapulmonary complications are presented in Table 3 and Supplementary Table 2. Tracheostomy was more often used in the older compared to the youngest patients. No differences in other pulmonary events were found. There was no effect of age on thrombotic complications, only the incidence of myocardial infarction was higher in the older age groups compared to the younger age groups. Acute kidney injury (AKI) occurred less often in the youngest age group compared to the older age groups, as was the need for renal replacement therapy.

### Outcomes

**Figure 2:**
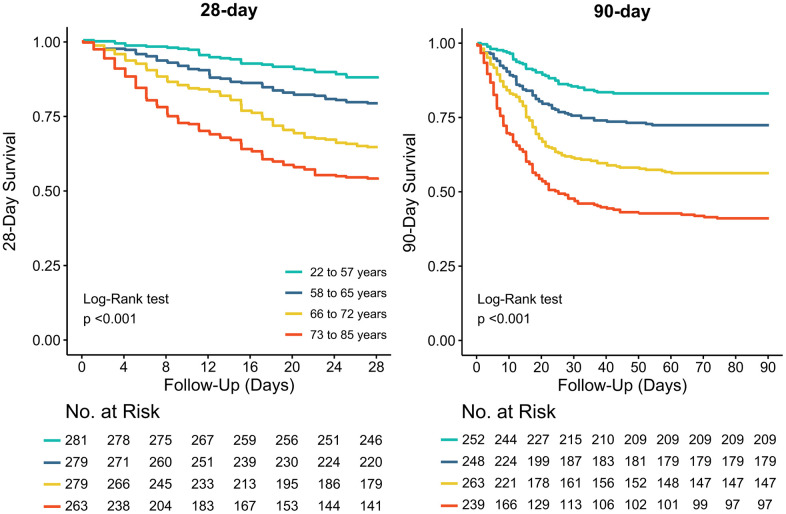
**Kaplan-Meier curves for 28-day and 90-day mortality per age group.** The Log-Rank test was used to calculate P values.

Patient outcomes are shown in Table 3, Supplementary Table 2 and Figure 2. In survivors, length of hospital and ICU stay increased while number of ventilator–free days decreased from the younger to the older age groups. Mortality rates increased from the lowest to the higher age group. After adjustment from effect modifiers, ICU– and hospital mortality, and 28– and 90–day were all higher in older patients (Supplementary Tables 3, 4).

## DISCUSSION

The results of this posthoc analysis of the PRoVENT–COVID study can be summarized as follows: (i.) there were no clinically meaningful differences in the key ventilator parameters between the 4 age groups; (ii.) on the first calendar day, mechanical power and peak pressure were lower in older patients but this effect disappeared in the succeeding days; (iii.) on the first four calendar days, EtCO_2_ was lower while PaCO_2_ was slightly higher in older patients; (iv) use of NMBAs was lower in older patients; (v) tracheostomy was more often used in older patients; (vi.) the incidence of AKI and the need for renal replacement therapy, and myocardial infarction was higher in older patients; (vii.) older patients stayed longer in the ICU and hospital; and (viii.) had higher mortality rates.

Our study has several strengths. The study included a large number of centers, both academic and non–academic, increasing the generalizability of the findings. Data were collected in a short time interval of 3 months, which minimizes the risk of changes in care over time. Data were collected by trained data collectors, which improved the quality of the data. Patients were followed until day 90, enabling for reporting on outcomes after stay in ICU. Of note, median age and other baseline characteristics are comparable to that in other studies [22, 23]. Also, in line with previous studies, the second and third age group had an evidently smaller range than the first and last age group, suggesting that middle–aged patients were the most prominent group admitted to the ICU.

Our findings suggest that ventilator management is not affected by age. Indeed, we found only minor, clinical meaningless, differences in key ventilator variables. The younger age groups had a higher BMI that could, at least in part, explain the higher median ΔP and Ppeak, and the higher mechanical power. Indeed, with a higher BMI higher thoracic pressures may be needed due to an increased stiffness of the chest wall [24]. Previous studies have shown higher EtCO_2_ values in older patients [25–27], but this was not seen in our cohort. Actually, the opposite relation between EtCO_2_ and age could be explained by the higher BMI in the younger age group, as an higher BMI may be associated with an increased production of carbon dioxide [28]. Of note, on the first day of mechanical ventilation, we did find a slightly higher PaCO_2_ but lower EtCO_2_ in older patients than in younger patients, but this difference disappeared in the following days. The age dependent reduction in body mass could also explain the lower use of NMBAs in older patients [29]. An association of higher age with lower use of NMBAs has been described before [30]. Other explanations for these differences include age–related differences in clearance of NMBAs, and maybe also the higher incidence of acute kidney injury (AKI) in older patients [29]. As AKI also affects clearance of opioids [31], the higher effective dose of opioids may have prevented use of NMBAs as well. Furthermore, physicians might be reluctance to use NMBAs in elderly patients because of the increased risk of prolonged immobility and thus ICU–acquired weakness [32].

Age is known to be a risk factor for complications like AKI, need for renal replacement therapy, and myocardial infarction [33–36]. Therefore, the increased incidence of these complications in older age groups was expected.

We found a strong association of age with mortality. This is, at least in part, in line with previous studies showing that age is a risk factor for mortality in invasively ventilated ICU patients in general [37–40], and in COVID–19 in particular [13, 41–43]. After adjusting for comorbidities and other effect modifiers, mortality rates remained significantly higher in the older patients. The 28–day mortality rate in our oldest age group was higher than that reported in a prospective study performed in elderly COVID–19 patients [44]. Interestingly, in that study it was shown that when patients were classified according to their frailty scale, mortality increased in vulnerable and frail patients. The level of frailty defines how vulnerable patients are for both physical and psychosocial factors. Frailty can be considered as a marker of biological age and, in addition to calendar age, can provide important prognostic information about clinical outcomes of ICU patients [44, 45]. Unfortunately, frailty was not, or incomplete reported in the medical records in the hospitals that participated in our study, but taken together the differences in mortality between our study and the previous study [44] suggest that patients in our cohort could have been frail more often.

In survivors, older patients stayed longer in the ICU and in the hospital, had a higher incidence of tracheostomy, and received ventilation for more days than younger patients. This may suggest that treatment discontinuation was not more common in elderly patients, but this could also be explained by the fact that older patients may have had already further disease progression or were in a higher need for supportive care. As data on treatment discontinuation were not collected in this analysis, this remains uncertain.

The findings of our study expand the current knowledge about the effects of age on ventilator management and outcomes in critically ill invasively ventilated COVID–19 patients. Lung–protective ventilation was well applied during the first COVID–19 outbreak, also in older patients. The higher mortality rates in older patients could help in decision–making about preventive measures. For example, these findings support guidelines to prioritize the elderly in vaccination programs. These insights may also further support a patient in deciding whether, and to what extent, ICU admission is still desirable.

Our analysis has several limitations. First, the question arises whether ‘door selection’ for ICU admission may has occurred. Particularly in the elderly, there is a possibility that ICU admission may no longer be considered beneficial if there is a relatively severe disease or premorbid functioning. Unfortunately, we could not collect data on ‘Do Not Resuscitate’ (DNR) codes or treatment discontinuation, e.g., withholding or withdrawal medical care in a reliable way. This cohort represents the first months of the pandemic in the Netherlands, during which an understandable emphasis was put on patient care rather than on reporting DNR codes in the patient records. However, since mortality is strongly influenced by the decision to discontinue treatment, this may have interfered with our findings [46]. Second, there is an intercountry difference in the willingness of patients to consider ICU admission. Compared to other countries, doctors as well as patients seem to be more reluctant to proceed with ICU admission when the situation worsens [47]. This could result in a selection bias and should be considered when extrapolating these results to other countries with a more liberal ICU admission policy. In fact, we expect the association of age with mortality to be even stronger in those countries. As mentioned above, we could also not collect data on the frailty, which is another important limitation. In addition, the PRoVENT–COVID trial was conducted in the first three months of the national outbreak in the Netherlands. Due to the introduction of e.g., dexamethasone and improved prophylaxis against venous thromboembolic events, and also the vaccination program, current ICU cohorts might be different.

## CONCLUSIONS

In this cohort of critically ill invasively ventilated COVID–19 patients, there were no meaningful differences in ventilator management between groups based on age quartiles. The use of adjunctive therapies for refractory hypoxemia was not affected by age, except for use of NMBAs that decreased with higher age. Older patients developed complications more often, had a longer duration of ventilation and higher mortality rates.

## Supplementary Material

PRoVENT –COVID Investigators

Supplementary Figures

Supplementary Tables

## References

[1] Medicine JHUa. COVID-19 Dashboard by the Center for Systems Science and Engineering. 2021. https://coronavirus.jhu.edu/map.html

[2] WHO. World Health Organization Coronavirus (COVID-19) Dashboard. 2021. https://covid19.who.int/

[3] Wiersinga WJ, Rhodes A, Cheng AC, Peacock SJ, Prescott HC. Pathophysiology, Transmission, Diagnosis, and Treatment of Coronavirus Disease 2019 (COVID-19): A Review. JAMA. 2020; 324:782–93. 10.1001/jama.2020.12839 32648899

[4] Sharma G, Goodwin J. Effect of aging on respiratory system physiology and immunology. Clin Interv Aging. 2006; 1:253–60. 10.2147/ciia.2006.1.3.253 18046878 PMC2695176

[5] Verbeken EK, Cauberghs M, Mertens I, Clement J, Lauweryns JM, Van de Woestijne KP. The senile lung. Comparison with normal and emphysematous lungs. 1. Structural aspects. Chest. 1992; 101:793–9. 10.1378/chest.101.3.793 1541148

[6] Gee MH, Gottlieb JE, Albertine KH, Kubis JM, Peters SP, Fish JE. Physiology of aging related to outcome in the adult respiratory distress syndrome. J Appl Physiol (1985). 1990; 69:822–9. 10.1152/jappl.1990.69.3.822 2246169

[7] Antonia Koutsoukou MK, Orfanos S, Rovina N, Dimitrakopoulou C, Kotanidou A, Koutsoukou A. ARDS in Aged Patients: Respiratory System Mechanics and Outcome. Health Sci J, 2017; 11:498 10.21767/1791-809X.1000498

[8] Salzberger B, Buder F, Lampl B, Ehrenstein B, Hitzenbichler F, Hanses F. Epidemiologie von SARS-CoV-2-Infektion und COVID-19 [Epidemiology of SARS-CoV-2 infection and COVID-19]. Internist (Berl). 2020; 61:782–8. 10.1007/s00108-020-00834-9 32548652 PMC7296906

[9] Yang J, Zheng Y, Gou X, Pu K, Chen Z, Guo Q, Ji R, Wang H, Wang Y, Zhou Y. Prevalence of comorbidities and its effects in patients infected with SARS-CoV-2: a systematic review and meta-analysis. Int J Infect Dis. 2020; 94:91–5. 10.1016/j.ijid.2020.03.017 32173574 PMC7194638

[10] Fang X, Li S, Yu H, Wang P, Zhang Y, Chen Z, Li Y, Cheng L, Li W, Jia H, Ma X. Epidemiological, comorbidity factors with severity and prognosis of COVID-19: a systematic review and meta-analysis. Aging (Albany NY). 2020; 12:12493–503. 10.18632/aging.103579 32658868 PMC7377860

[11] Chen T, Wu D, Chen H, Yan W, Yang D, Chen G, Ma K, Xu D, Yu H, Wang H, Wang T, Guo W, Chen J, et al. Clinical characteristics of 113 deceased patients with coronavirus disease 2019: retrospective study. BMJ. 2020; 368:m1091. 10.1136/bmj.m1091 32217556 PMC7190011

[12] Wu Z, McGoogan JM. Characteristics of and Important Lessons From the Coronavirus Disease 2019 (COVID-19) Outbreak in China: Summary of a Report of 72 314 Cases From the Chinese Center for Disease Control and Prevention. JAMA. 2020; 323:1239–42. 10.1001/jama.2020.2648 32091533

[13] Grasselli G, Greco M, Zanella A, Albano G, Antonelli M, Bellani G, Bonanomi E, Cabrini L, Carlesso E, Castelli G, Cattaneo S, Cereda D, Colombo S, et al, and COVID-19 Lombardy ICU Network. Risk Factors Associated With Mortality Among Patients With COVID-19 in Intensive Care Units in Lombardy, Italy. JAMA Intern Med. 2020; 180:1345–55. 10.1001/jamainternmed.2020.3539 32667669 PMC7364371

[14] Tinetti ME, Fried TR, Boyd CM. Designing health care for the most common chronic condition--multimorbidity. JAMA. 2012; 307:2493–4. 10.1001/jama.2012.5265 22797447 PMC4083627

[15] Hirani V, Naganathan V, Blyth F, Le Couteur DG, Gnjidic D, Stanaway FF, Seibel MJ, Waite LM, Handelsman DJ, Cumming RG. Multiple, but not traditional risk factors predict mortality in older people: the Concord Health and Ageing in Men Project. Age (Dordr). 2014; 36:9732. 10.1007/s11357-014-9732-2 25403157 PMC4234745

[16] Macaulay R, Akbar AN, Henson SM. The role of the T cell in age-related inflammation. Age (Dordr). 2013; 35:563–72. 10.1007/s11357-012-9381-2 22252437 PMC3636399

[17] Hoogendijk EO, Afilalo J, Ensrud KE, Kowal P, Onder G, Fried LP. Frailty: implications for clinical practice and public health. Lancet. 2019; 394:1365–75. 10.1016/S0140-6736(19)31786-6 31609228

[18] Botta M, Tsonas AM, Pillay J, Boers LS, Algera AG, Bos LD, Dongelmans DA, Hollmann MW, Horn J, Vlaar AP, Schultz MJ, Neto AS, Paulus F, and PRoVENT-COVID Collaborative Group. Ventilation management and clinical outcomes in invasively ventilated patients with COVID-19 (PRoVENT-COVID): a national, multicentre, observational cohort study. Lancet Respir Med. 2021; 9:139–48. 10.1016/S2213-2600(20)30459-8 33169671 PMC7584441

[19] Boers NS, Botta M, Tsonas AM, Algera AG, Pillay J, Dongelmans DA, Horn J, Vlaar AP, Hollmann MW, Bos LD, Paulus F, Neto AS, Schultz MJ, and PRoVENT-COVID investigators†. PRactice of VENTilation in Patients with Novel Coronavirus Disease (PRoVENT-COVID): rationale and protocol for a national multicenter observational study in The Netherlands. Ann Transl Med. 2020; 8:1251. 10.21037/atm-20-5107 33178783 PMC7607125

[20] Ranieri VM, Rubenfeld GD, Thompson BT, Ferguson ND, Caldwell E, Fan E, Camporota L, Slutsky AS, and ARDS Definition Task Force. Acute respiratory distress syndrome: the Berlin Definition. JAMA. 2012; 307:2526–33. 10.1001/jama.2012.5669 22797452

[21] Schavemaker R, Schultz MJ, Lagrand WK, van Slobbe-Bijlsma ER, Serpa Neto A, Paulus F, and The PRoVENT-Covid Collaborative Group. Associations of Body Mass Index with Ventilation Management and Clinical Outcomes in Invasively Ventilated Patients with ARDS Related to COVID-19-Insights from the PRoVENT-COVID Study. J Clin Med. 2021; 10:1176. 10.3390/jcm10061176 33799735 PMC8000207

[22] COVID-ICU Group on behalf of the REVA Network and the COVID-ICU Investigators. Clinical characteristics and day-90 outcomes of 4244 critically ill adults with COVID-19: a prospective cohort study. Intensive Care Med. 2021; 47:60–73. 10.1007/s00134-020-06294-x 33211135 PMC7674575

[23] Richards-Belle A, Orzechowska I, Gould DW, Thomas K, Doidge JC, Mouncey PR, Christian MD, Shankar-Hari M, Harrison DA, Rowan KM, and ICNARC COVID-19 Team. COVID-19 in critical care: epidemiology of the first epidemic wave across England, Wales and Northern Ireland. Intensive Care Med. 2020; 46:2035–47. 10.1007/s00134-020-06267-0 33034689 PMC7545019

[24] Slutsky AS, Ranieri VM. Ventilator-induced lung injury. N Engl J Med. 2013; 369:2126–36. 10.1056/NEJMra1208707 24283226

[25] Nunn JF, Hill DW. Respiratory dead space and arterial to end-tidal carbon dioxide tension difference in anesthetized man. J Appl Physiol. 1960; 15:383–9. 10.1152/jappl.1960.15.3.383 14427915

[26] St Croix CM, Cunningham DA, Kowalchuk JM, McConnell AK, Kirby AS, Scheuermann BW, Petrella RJ, Paterson DH. Estimation of arterial PCO2 in the elderly. J Appl Physiol (1985). 1995; 79:2086–93. 10.1152/jappl.1995.79.6.2086 8847277

[27] Satoh K, Ohashi A, Kumagai M, Sato M, Kuji A, Joh S. Evaluation of Differences between PaCO_2_ and ETCO_2_ by Age as Measured during General Anesthesia with Patients in a Supine Position. Journal of Anesthesiology. 2015; 2015:710537. 10.1155/2015/710537

[28] De Jong A, Chanques G, Jaber S. Mechanical ventilation in obese ICU patients: from intubation to extubation. Crit Care. 2017; 21:63. 10.1186/s13054-017-1641-1 28320439 PMC5359820

[29] Lee LA, Athanassoglou V, Pandit JJ. Neuromuscular blockade in the elderly patient. J Pain Res. 2016; 9:437–44. 10.2147/JPR.S85183 27382330 PMC4918890

[30] Arroliga AC, Thompson BT, Ancukiewicz M, Gonzales JP, Guntupalli KK, Park PK, Wiedemann HP, Anzueto A, and Acute Respiratory Distress Syndrome Network. Use of sedatives, opioids, and neuromuscular blocking agents in patients with acute lung injury and acute respiratory distress syndrome. Crit Care Med. 2008; 36:1083–8. 10.1097/CCM.0B013E3181653895 18401254

[31] Ball M, Moore RA, Fisher A, McQuay HJ, Allen MC, Sear J. Renal failure and the use of morphine in intensive care. The Lancet. 1985; 325:784–6. 10.1016/S0140-6736(85)91448-5 2858668

[32] deBacker J, Hart N, Fan E. Neuromuscular Blockade in the 21st Century Management of the Critically Ill Patient. Chest. 2017; 151:697–706. 10.1016/j.chest.2016.10.040 27818334

[33] Dhingra R, Vasan RS. Age as a risk factor. Med Clin North Am. 2012; 96:87–91. 10.1016/j.mcna.2011.11.003 22391253 PMC3297980

[34] Rodgers JL, Jones J, Bolleddu SI, Vanthenapalli S, Rodgers LE, Shah K, Karia K, Panguluri SK. Cardiovascular Risks Associated with Gender and Aging. J Cardiovasc Dev Dis. 2019; 6:19. 10.3390/jcdd6020019 31035613 PMC6616540

[35] Chao CT, Wang J, Wu HY, Huang JW, Chien KL. Age modifies the risk factor profiles for acute kidney injury among recently diagnosed type 2 diabetic patients: a population-based study. Geroscience. 2018; 40:201–17. 10.1007/s11357-018-0013-3 29488059 PMC5964062

[36] Schmitt R, Coca S, Kanbay M, Tinetti ME, Cantley LG, Parikh CR. Recovery of kidney function after acute kidney injury in the elderly: a systematic review and meta-analysis. Am J Kidney Dis. 2008; 52:262–71. 10.1053/j.ajkd.2008.03.005 18511164

[37] Ely EW, Wheeler AP, Thompson BT, Ancukiewicz M, Steinberg KP, Bernard GR. Recovery rate and prognosis in older persons who develop acute lung injury and the acute respiratory distress syndrome. Ann Intern Med. 2002; 136:25–36. 10.7326/0003-4819-136-1-200201010-00007 11777361

[38] Brun-Buisson C, Minelli C, Bertolini G, Brazzi L, Pimentel J, Lewandowski K, Bion J, Romand JA, Villar J, Thorsteinsson A, Damas P, Armaganidis A, Lemaire F, and ALIVE Study Group. Epidemiology and outcome of acute lung injury in European intensive care units. Results from the ALIVE study. Intensive Care Med. 2004; 30:51–61. 10.1007/s00134-003-2022-6 14569423

[39] Quality of Life After Mechanized Ventilation in the Elderly Study Investigators. 2-month mortality and functional status of critically ill adult patients receiving prolonged mechanical ventilation. Chest. 2002; 121:549–58. 10.1378/chest.121.2.549 11834671

[40] Santa Cruz R, Villarejo F, Figueroa A, Cortés-Jofré M, Gagliardi J, Navarrete M. Mortality in Critically Ill Elderly Individuals Receiving Mechanical Ventilation. Respir Care. 2019; 64:473–83. 10.4187/respcare.06586 30944228

[41] Jung C, Fjølner J, Bruno RR, Wernly B, Artigas A, Bollen Pinto B, Schefold JC, Wolff G, Kelm M, Beil M, Sviri S, van Heerden PV, Szczeklik W, et al, and COVIP Study Group. Differences in mortality in critically ill elderly patients during the second COVID-19 surge in Europe. Crit Care. 2021; 25:344. 10.1186/s13054-021-03739-7 34556171 PMC8459701

[42] Leoni ML, Lombardelli L, Colombi D, Bignami EG, Pergolotti B, Repetti F, Villani M, Bellini V, Rossi T, Halasz G, Caprioli S, Micheli F, Nolli M. Prediction of 28-day mortality in critically ill patients with COVID-19: Development and internal validation of a clinical prediction model. PLoS One. 2021; 16:e0254550. 10.1371/journal.pone.0254550 34255793 PMC8277063

[43] Guillon A, Laurent E, Godillon L, Kimmoun A, Grammatico-Guillon L. Long-term mortality of elderly patients after intensive care unit admission for COVID-19. Intensive Care Med. 2021; 47:710–2. 10.1007/s00134-021-06399-x 33844045 PMC8040757

[44] Jung C, Flaatten H, Fjølner J, Bruno RR, Wernly B, Artigas A, Bollen Pinto B, Schefold JC, Wolff G, Kelm M, Beil M, Sviri S, van Heerden PV, et al, and COVIP study group. The impact of frailty on survival in elderly intensive care patients with COVID-19: the COVIP study. Crit Care. 2021; 25:149. 10.1186/s13054-021-03551-3 33874987 PMC8054503

[45] Flaatten H, de Lange DW, Artigas A, Bin D, Moreno R, Christensen S, Joynt GM, Bagshaw SM, Sprung CL, Benoit D, Soares M, Guidet B. The status of intensive care medicine research and a future agenda for very old patients in the ICU. Intensive Care Med. 2017; 43:1319–28. 10.1007/s00134-017-4718-z 28238055

[46] Flaatten H, Guidet B, de Lange DW, Beil M, Leaver SK, Fjølner J, van Heerden PV, Sigal S, Szczeklik W, Jung C. The importance of revealing data on limitation of life sustaining therapy in critical ill elderly Covid-19 patients. J Crit Care. 2022; 67:147–8. 10.1016/j.jcrc.2021.10.024 34781100 PMC8588784

[47] Haas LE, Karakus A, Holman R, Cihangir S, Reidinga AC, de Keizer NF. Trends in hospital and intensive care admissions in the Netherlands attributable to the very elderly in an ageing population. Crit Care. 2015; 19:353. 10.1186/s13054-015-1061-z 26423744 PMC4588268

