# Supplementary material for: The effect of age on ventilation management and clinical outcomes in critically ill COVID–19 patients––insights from the PRoVENT–COVID study
Source: Aging (Albany NY). 2022 Jan 31;14(3):1087–109. doi: 10.18632/aging.203863 (PMC8876900; doi:10.18632/aging.203863)
Supplement: PRoVENT –COVID Investigators [file aging-14-203863-s001.pdf]

## PROVENT–COVID INVESTIGATORS

Investigators: (in alphabetic order) J.P. van Akkeren[7]; A.G. Algera[1]; C.K. Algoe[1]; R.B. van Amstel[1]; O.L. Baur[1]; P. van de Berg[8]; A.E. van den Berg[9]; D.C.J.J. Bergmans[10]; D.I. van den Bersselaar[1]; F.A. Bertens[1]; A.J.G.H. Bindels[8]; M.M. de Boer[1]; S. den Boer[11]; L.S. Boers[1]; M. Bogerd[1]; L.D.J. Bos[1]; M. Botta[1]; J.S. Breel[2]; H. de Bruin[1]; S. de Bruin[1]; C.L. Bruna[1]; L.A. Buiteman–Kruizinga[12]; O. L. Cremer[13]; R.M. Determann[14]; W. Dieperink[15]; D.A. Dongelmans[1]; H.S. Franke[15]; M.S. Galek -Aldridge[1]; M.J. de Graaff[16]; L.A. Hagens[1]; J.J. Haringman[17]; S.T. van der Heide[1]; P.L.J. van der Heiden[12]; N.F.L. Heijnen[10]; S.J.P. Hiel[7]; L.L. Hoeijmakers[1]; L. Hol[1,2]; M.W. Hollmann[2]; M.E. Hoogendoorn[17]; J. Horn[1]; R. P. van der Horst[18]; E.L.K. Ie[1]; D. Ivanov[1]; N.P. Juffermans[14]; E. Kho[1]; E.S. de Klerk[2]; A.W.M.M. Koopman-van Gemert[19]; M. Koopmans[14]; S. Kucukcelebi[1]; M.A. Kuiper[20]; D.W. de Lange[13]; N. van Mourik[1]; S.G. Nijbroek[1,2]; M. Onrust[15]; E.A.N. Oostdijk[21]; F. Paulus[1,3]; C.J. Pennartz[1]; J. Pillay[1,15]; L. Pisani[1]; I.M. Purmer[22]; T.C.D. Rettig[23]; J.P. Roozeman[1]; M.T.U. Schuijt[1]; M.J. Schultz[1,5,6]; A. Serpa Neto[4]; M.E. Sleeswijk[24]; M.R. Smit[1]; P.E. Spronk[25]; W. Stilma[1]; A.C. Strang[26]; A.M. Tsonas[1]; P.R. Tuinman[27]; C.M.A. Valk[1]; F.L. Veen-Schra[17]; L.I. Veldhuis[1]; P. van Velzen[9]; W.H. van der Ven[2]; A.P.J. Vlaar[1]; P. van Vliet[28]; P.H.J. van der Voort[15]; L. van Welie[29]; H.J.F.T. Wesselink[17]; H.H. van der Wier-Lubbers[17]; B. van Wijk[1]; T. Winters[1]; W.Y. Wong[1]; A.R.H. van Zanten[29].

Affiliations: Amsterdam University Medical Centers, location ‘Academic Medical Center’, Amsterdam, The Netherlands: [1]Department of Intensive Care, [2]Department of Anesthesiology; Amsterdam University of Applied Sciences, Faculty of Health, Amsterdam, The Netherlands: [3]ACHIEVE, Centre of Applied Research; Hospital Israelita Albert Einstein; São Paulo, Brazil: [4]Department of Critical Care Medicine; Mahidol University, Bangkok, Thailand: [5]Mahidol–Oxford Tropical Medicine Research Unit (MORU); University of Oxford, Oxford, United Kingdom: [6]Nuffield Department of Medicine; Maxima Medical Center, Eindhoven, The Netherlands: [7]Department of Intensive Care; Catharina Hospital, Eindhoven, The Netherlands: [8]Department of Intensive Care; Dijklander Hospital, location Hoorn, Hoorn, The Netherlands: [9]Department of Intensive Care; Maastricht University Medical Center, Maastricht, The Netherlands: [10]Department of Intensive Care; Spaarne Hospital, Haarlem, The Netherlands: [11]Department of Intensive Care; Reinier de Graaf Hospital, Delft, The Netherlands: [12]Department of Intensive Care; University Medical Center Utrecht, Utrecht, The Netherlands: [13]Department of Intensive Care; OLVG hospital, location East, Amsterdam, The Netherlands: [14]Department of Intensive Care; University Medical Center Groningen, Groningen, The Netherlands: [15]Department of Intensive Care; Sint Antonius Hospital, Nieuwegein, The Netherlands: [16]Department of Intensive Care; Isala Hospital, Zwolle, The Netherlands: [17]Department of Intensive Care; Zuyderland Hospital, Heerlen and Sittard, The Netherlands: [18]Department of Intensive Care; ZGT Hospital, Almelo, The Netherlands: [19]Department of Intensive Care; Medical Center Leeuwarden, Leeuwarden, The Netherlands: [20]Department of Intensive Care; Maasstad Hospital, Rotterdam, The Netherlands: [21]Department of Intensive Care; Haga Hospital, the Hague, The Netherlands: [22]Department of Intensive Care; Amphia Hospital, Breda, The Netherlands: [23]Department of Intensive Care; Flevo Hospital, Almere, The Netherlands: [24]Department of Intensive Care; Gelre Hospital, Apeldoorn and Zutphen, The Netherlands: [25]Department of Intensive Care; Rijnstate Hospital, Arnhem, The Netherlands: [26]Department of Intensive Care; Amsterdam University Medical Centers, location ‘VU Medical Center’, Amsterdam, The Netherlands: [27]Department of Intensive Care; Haaglanden Medical Center, location Westeinde, Hague, The Netherlands: [28]Department of Intensive Care; Gelderse Vallei Hospital, Ede, The Netherlands: [29]Department of Intensive Care.
