# Supplementary material for: The effect of age on ventilation management and clinical outcomes in critically ill COVID–19 patients––insights from the PRoVENT–COVID study
Source: Aging (Albany NY). 2022 Jan 31;14(3):1087–109. doi: 10.18632/aging.203863 (PMC8876900; doi:10.18632/aging.203863)
Supplement: Supplementary Figures [file aging-14-203863-s002.pdf]

SUPPLEMENTARY FIGURES

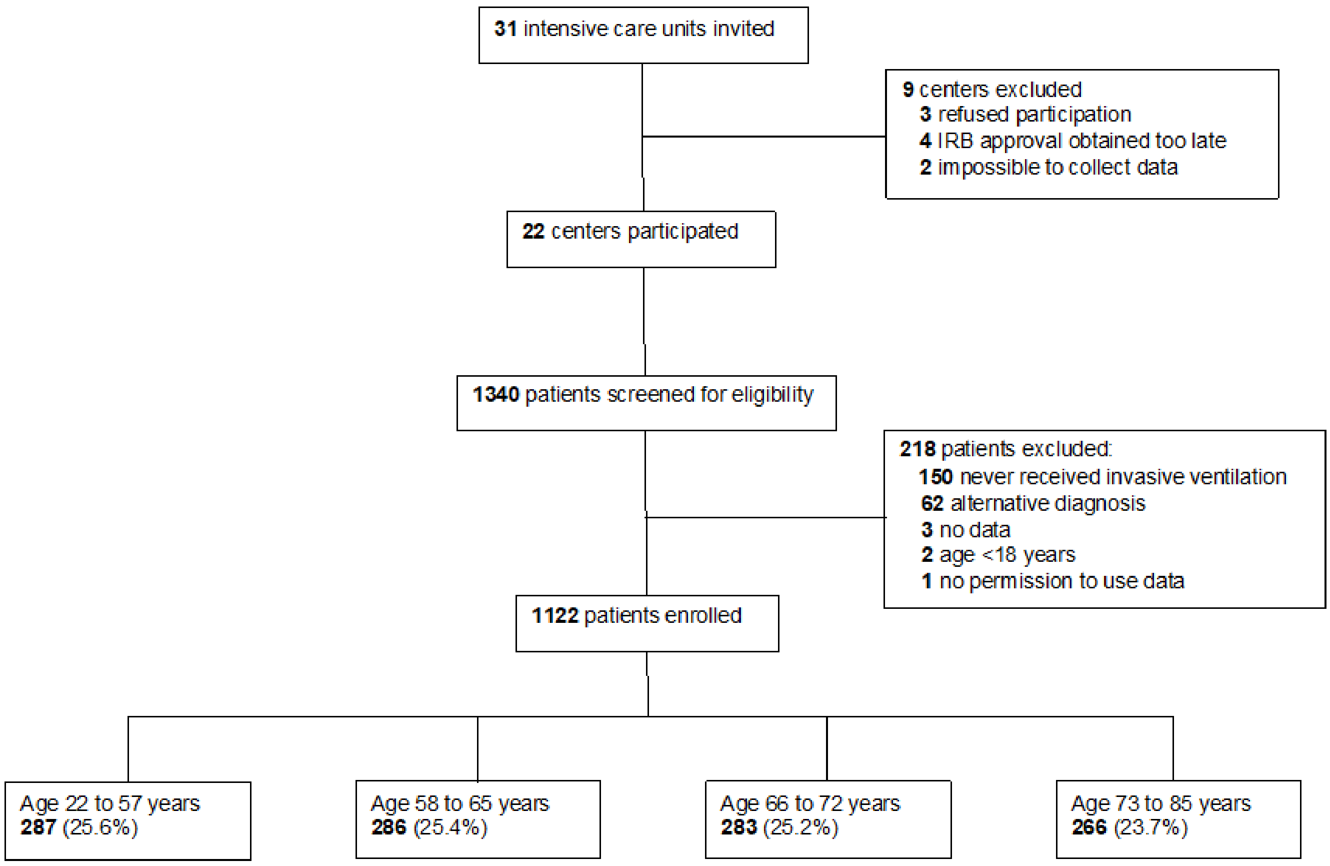

Supplementary Figure 1. CONSORT flow chart of study population.

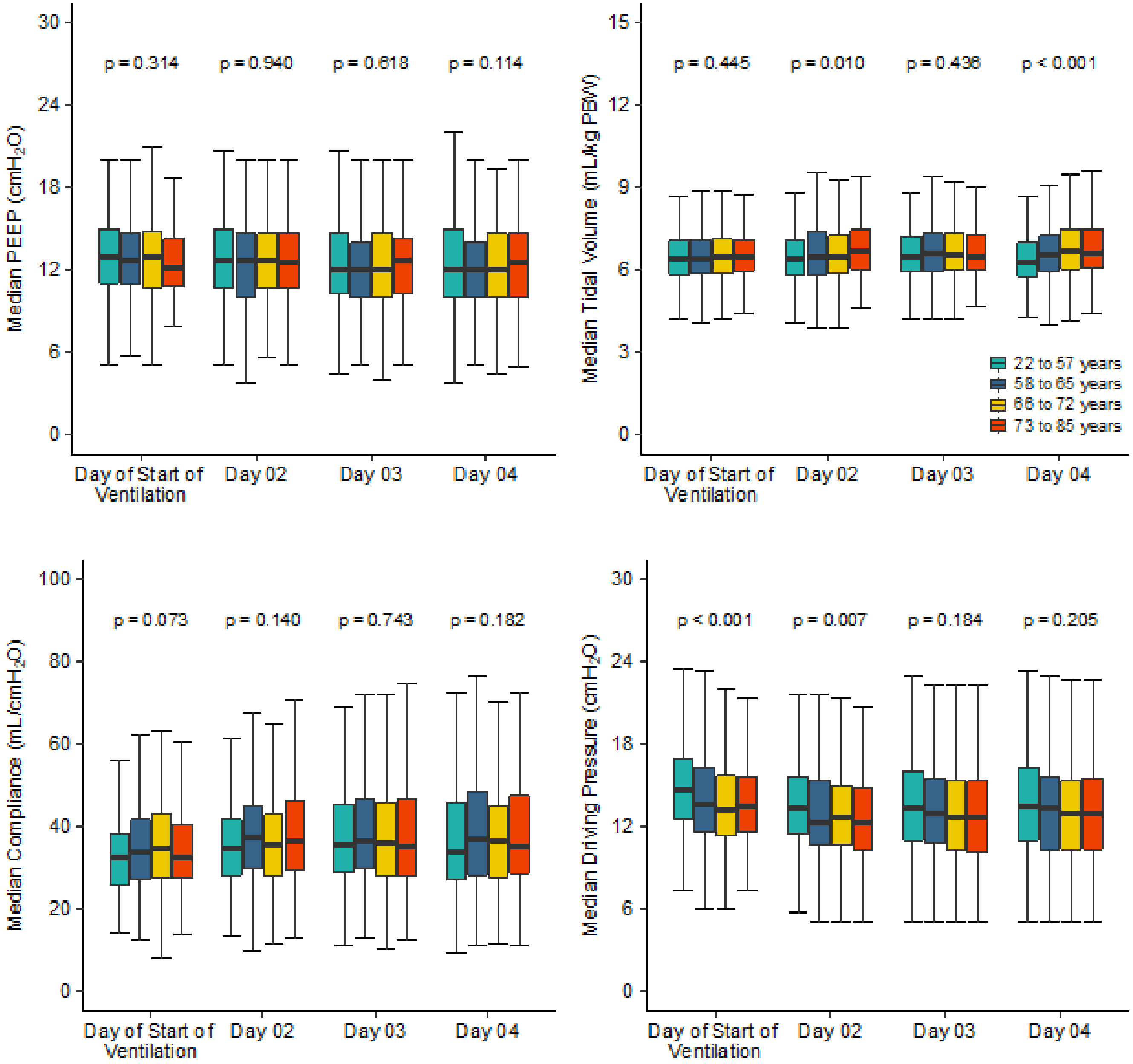

Supplementary Figure 2. Ventilatory variables in the first four days of ventilation. Boxes represent median and interquartile range. Median was calculated from the mean value of three or four measurements available on each day of ventilation. The Kruskal-Wallis test is used to calculate p-values.

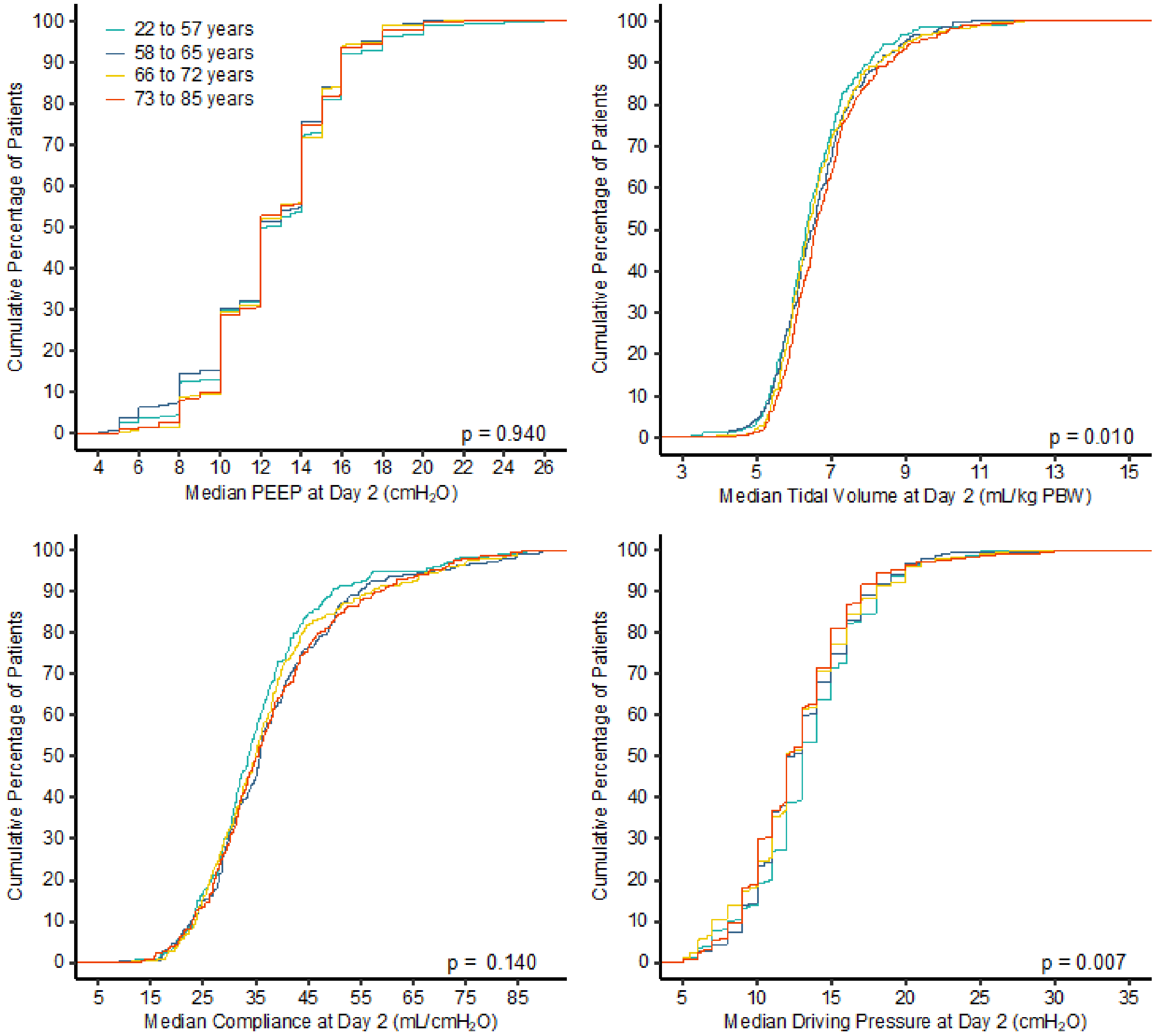

Supplementary Figure 3. Cumulative frequency distribution of median PEEP to tidal volume to compliance and driving pressure on the second day of ventilation. Mean values were calculated from three measurements available on the second day of ventilation. The Kruskal-Wallis test is used to calculate p-values.

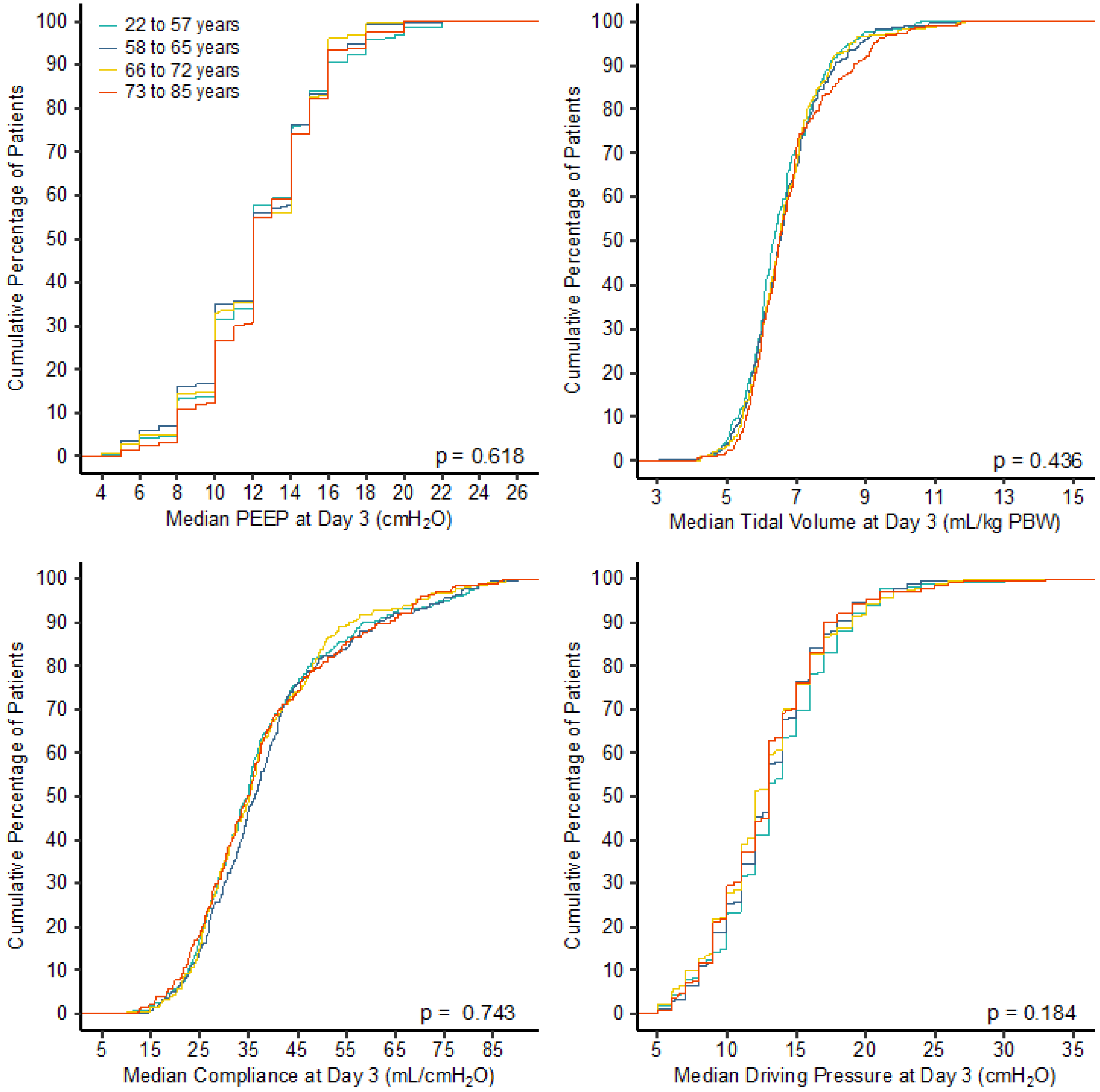

Supplementary Figure 4. Cumulative frequency distribution of median PEEP to tidal volume to compliance and driving pressure on the third day of ventilation. Mean values were calculated from three measurements available on the third day of ventilation. The Kruskal-Wallis test is used to calculate p-values.

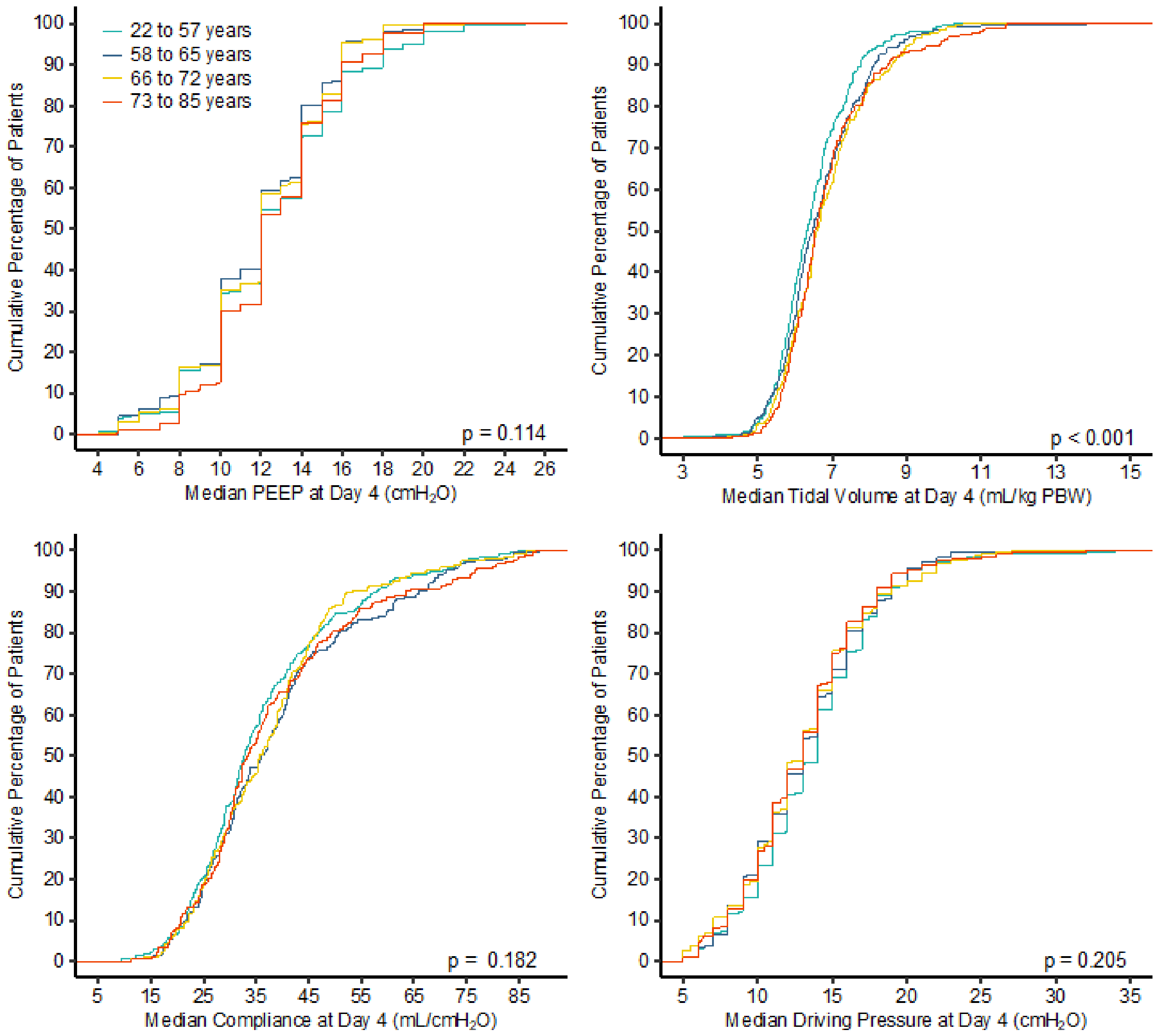

Supplementary Figure 5. Cumulative frequency distribution of median PEEP to tidal volume to compliance and driving pressure on the fourth day of ventilation. Mean values were calculated from three measurements available on the fourth day of ventilation. The Kruskal-Wallis test was is to calculate p-values.
