# Supplementary material for: The effect of age on ventilation management and clinical outcomes in critically ill COVID–19 patients––insights from the PRoVENT–COVID study
Source: Aging (Albany NY). 2022 Jan 31;14(3):1087–109. doi: 10.18632/aging.203863 (PMC8876900; doi:10.18632/aging.203863)
Supplement: Supplementary Tables [file aging-14-203863-s003.pdf]

SUPPLEMENTARY TABLES

Supplementary Table 1. Characteristics of mechanical ventilation in the first four days of ventilation.

	Age 22 to 57 years (*n* = 287)	Age 58 to 65 years (*n* = 286)	Age 66 to 72 years (*n* = 283)	Age 73 to 85 years (*n* = 266)	*P* value
Tidal volume, mL/kg PBW					
Day 1	6.4 [5.8 to 7.0]	6.4 [5.9 to 7.1]	6.5 [5.9 to 7.1]	6.5 [6.0 to 7.1]	0.445
Day 2	6.4 [5.8 to 7.1]	6.5 [5.8 to 7.4]	6.5 [5.9 to 7.3]	6.7 [6.0 to 7.4]	0.010
Day 3	6.5 [5.9 to 7.2]	6.6 [5.9 to 7.3]	6.5 [6.0 to 7.4]	6.5 [6.0 to 7.2]	0.437
Day 4	6.3 [5.8 to 7.0]	6.6 [5.9 to 7.2]	6.7 [6.0 to 7.5]	6.6 [6.1 to 7.5]	<0.001
PEEP, cmH_2O					
Day 1	13.0 [11.0 to 15.0]	12.7 [11.0 to 14.6]	13.0 [10.7 to 14.8]	12.2 [10.8 to 14.2]	0.314
Day 2	12.7 [10.7 to 15.0]	12.7 [10.0 to 14.7]	12.7 [10.7 to 14.7]	12.6 [10.7 to 14.7]	0.940
Day 3	12.0 [10.3 to 14.7]	12.0 [10.0 to 14.0]	12.0 [10.0 to 14.7]	12.7 [10.3 to 14.3]	0.618
Day 4	12.0 [10.0 to 15.0]	12.0 [10.0 to 14.0]	12.0 [10.0 to 14.7]	12.5 [10.0 to 14.7]	0.114
Driving pressure, cmH_2O					
Day 1	14.7 [12.5 to 17.0]	13.8 [11.7 to 16.3]	13.2 [11.3 to 15.7]	13.5 [11.6 to 15.7]	<0.001
Day 2	13.3 [11.4 to 15.7]	12.3 [10.7 to 15.3]	12.7 [10.7 to 15.0]	12.4 [10.3 to 15.0]	0.007
Day 3	13.3 [11.0 to 16.0]	13.0 [10.8 to 15.5]	12.7 [10.3 to 15.3]	12.7 [10.1 to 15.3]	0.184
Day 4	13.7 [11.0 to 16.3]	13.3 [10.3 to 15.7]	13.0 [10.3 to 15.3]	13.0 [10.3 to 15.5]	0.205
Compliance, mL/cmH_2O					
Day 1	32.4 [25.9 to 38.3]	33.8 [27.1 to 41.7]	34.7 [27.7 to 43.3]	32.6 [27.3 to 40.7]	0.073
Day 2	34.9 [28.4 to 42.2]	37.5 [29.8 to 45.4]	35.7 [28.3 to 43.5]	36.6 [29.6 to 46.7]	0.140
Day 3	35.5 [28.9 to 45.4]	36.5 [29.6 to 47.0]	36.0 [28.2 to 47.1]	35.4 [27.9 to 47.5]	0.743
Day 4	33.9 [26.9 to 45.6]	36.8 [28.6 to 49.1]	37.0 [27.9 to 47.0]	35.3 [28.7 to 49.4]	0.182
Peak pressure, cmH_2O					
Day 1	27.7 [25.0 to 30.8]	26.7 [23.3 to 30.0]	26.0 [23.3 to 29.2]	26.2 [23.6 to 29.0]	<0.001
Day 2	26.3 [23.0 to 29.7]	25.3 [22.3 to 29.0]	25.7 [22.0 to 28.3]	25.3 [22.0 to 28.3]	0.102
Day 3	26.0 [22.0 to 29.7]	25.7 [21.3 to 28.5]	25.3 [20.7 to 28.8]	25.3 [21.3 to 29.0]	0.362
Day 4	26.3 [22.0 to 29.7]	25.3 [20.4 to 28.9]	25.3 [20.7 to 28.7]	25.3 [22.0 to 29.3]	0.145
Mechanical power, J/min					
Day 1	19.2 [16.0 to 23.7]	19.3 [15.9 to 23.1]	17.9 [14.7 to 22.3]	17.2 [14.6 to 20.9]	<0.001
Day 2	18.8 [15.7 to 23.5]	19.1 [15.8 to 23.2]	18.6 [14.6 to 22.9]	18.1 [14.4 to 22.3]	0.237
Day 3	19.2 [15.1 to 24.1]	19.7 [15.4 to 23.8]	18.8 [14.9 to 22.6]	18.7 [15.2 to 23.1]	0.619
Day 4	19.2 [15.9 to 24.0]	19.5 [15.2 to 23.9]	19.3 [15.1 to 23.3]	19.3 [16.3 to 23.5]	0.882
$PaCO_2$, mmHg					
Day 1	42.9 [38.3 to 48.4]	44.6 [39.8 to 49.5]	46.1 [39.9 to 52.0]	45.0 [39.1 to 50.9]	0.002
Day 2	44.5 [40.0 to 49.5]	46.6 [41.8 to 52.5]	45.4 [42.0 to 53.3]	45.5 [40.6 to 51.8]	0.060
Day 3	46.8 [42.5 to 54.8]	48.3 [43.4 to 53.8]	47.3 [42.8 to 55.3]	47.3 [41.8 to 54.0]	0.483
Day 4	48.5 [43.3 to 55.3]	49.3 [44.5 to 54.3]	48.8 [43.8 to 56.0]	48.6 [42.5 to 54.3]	0.724
$EtCO_2$, mmHg					
Day 1	38.0 [33.8 to 43.8]	37.7 [33.3 to 42.8]	36.3 [31.9 to 42.0]	35.3 [31.6 to 39.9]	<0.001
Day 2	39.8 [35.5 to 44.3]	38.6 [34.8 to 44.3]	36.8 [32.2 to 41.3]	36.8 [31.8 to 41.4]	<0.001
Day 3	41.0 [36.3 to 46.5]	38.8 [34.5 to 43.0]	37.5 [33.3 to 42.5]	36.5 [32.8 to 42.7]	<0.001
Day 4	42.3 [37.0 to 49.0]	38.5 [35.0 to 44.3]	37.5 [32.2 to 42.8]	37.5 [33.0 to 42.5]	<0.001
FiO_2					
Day 1	0.6 [0.5 to 0.7]	0.6 [0.5 to 0.7]	0.6 [0.5 to 0.7]	0.6 [0.5 to 0.7]	0.286
Day 2	0.4 [0.4 to 0.5]	0.4 [0.4 to 0.5]	0.4 [0.4 to 0.5]	0.5 [0.4 to 0.5]	0.269
Day 3	0.4 [0.4 to 0.5]	0.4 [0.4 to 0.5]	0.4 [0.4 to 0.5]	0.4 [0.4 to 0.5]	0.750
Day 4	0.4 [0.4 to 0.5]	0.4 [0.4 to 0.5]	0.5 [0.4 to 0.6]	0.5 [0.4 to 0.6]	0.294
PaO_2, mmHg					
Day 1	81.0 [71.5 to 99.3]	78.7 [71.3 to 93.4]	82.4 [72.7 to 95.4]	83.3 [75.0 to 96.0]	0.018
Day 2	75.0 [69.3 to 86.3]	75.3 [69.1 to 84.7]	75.5 [69.8 to 84.5]	76.5 [69.7 to 84.5]	0.782
Day 3	72.5 [67.1 to 82.1]	72.3 [66.0 to 80.8]	74.5 [67.4 to 81.3]	73.8 [67.5 to 81.0]	0.443
Day 4	72.0 [66.0 to 80.3]	70.8 [64.9 to 78.3]	72.3 [66.1 to 79.3]	73.1 [68.0 to 80.3]	0.120

Supplementary Table 2. Posthoc dunn test for paired comparison for patient outcomes.

**Tracheostomy**		**Age 22 to 57 years**	**Age 58 to 65 years**	**Age 66 to 72 years**
Age 58 to 65 years	Z test statistic	-2.992		
	P value	0.008		
Age 66 to 72 years	Z test statistic	-1.504	1.478	
	P value	0.397	0.148	
Age 73 to 85 years	Z test statistic	-1.433	1.508	0.050
	P value	0.455	0.395	1.000
**Myocardial infarction**		**Age 22 to 57 years**	**Age 58 to 65 years**	**Age 66 to 72 years**
Age 58 to 65 years	Z test statistic	0.703		
	P value	1.000		
Age 66 to 72 years	Z test statistic	-1.788	-2.487	
	P value	0.221	0.039	
Age 73 to 85 years	Z test statistic	-1.916	-2.605	-0.156
	P value	0.166	0.028	1.000
**Acute Kidney injury**		**Age 22 to 57 years**	**Age 58 to 65 years**	**Age 66 to 72 years**
Age 58 to 65 years	Z test statistic	-4.358		
	P value	<0.001		
Age 66 to 72 years	Z test statistic	-3.315	1.025	
	P value	0.003	0.916	
Age 73 to 85 years	Z test statistic	-5.242	-0.963	-1.966
	P value	<0.001	1.000	0.148
**Need for renal replacement therapy**		**Age 22 to 57 years**	**Age 58 to 65 years**	**Age 66 to 72 years**
Age 58 to 65 years	Z test statistic	-2.936		
	P value	0.010		
Age 66 to 72 years	Z test statistic	-2.454	0.474	
	P value	0.042	1.000	
Age 73 to 85 years	Z test statistic	-2.121	0.761	0.293
	P value	0.102	1.000	1.000
**Use of neuromuscular blocking agents**		**Age 22 to 57 years**	**Age 58 to 65 years**	**Age 66 to 72 years**
Age 58 to 65 years	Z test statistic	1.881		
	P value	0.180		
Age 66 to 72 years	Z test statistic	1.083	-0.792	
	P value	0.837	1.000	
Age 73 to 85 years	Z test statistic	3.588	1.740	2.514
	P value	0.001	0.246	0.036
**Ventilator-free days at day 28**		**Age 22 to 57 years**	**Age 58 to 65 years**	**Age 66 to 72 years**
Age 58 to 65 years	Z test statistic	4.488		
	P value	<0.001		
Age 66 to 72 years	Z test statistic	6.9400	2.446	
	P value	<0.001	0.043	
Age 73 to 85 years	Z test statistic	9.309	4.855	2.435
	P value	<0.001	<0.001	0.045

Supplementary Table 3. Multivariable assessment of factors associated with 28-day and 90-day mortality.

	28-day mortality		90-day mortality	
	**Hazard ratio (95% CI)**	**P value**	**Hazard ratio (95% CI)**	**P value**
**Age category**				
Age 22 to 57 years	1 (reference)		1 (reference)	
Age 58 to 65 years	1.37 (0.89 to 2.11)	0.150	1.49 (1.00 to 2.23)	0.050
Age 66 to 72 years	2.16 (1.43 to 3.25)	<0.001	2.32 (1.59 to 3.40)	<0.001
Age 73 to 85 years	3.35 (2.24 to 5.01)	<0.001	4.05 (2.77 to 5.93)	<0.001
**Demographic characteristics**				
Male gender	1.16 (0.88 to 1.52)	0.290	1.25 (0.96 to 1.62)	0.093
Body-mass index to kg/m^2	0.97 (0.85 to 1.10)	0.630	1.00 (0.90 to 1.11)	0.980
Hypertension	1.32 (1.02 to 1.72)	0.038	1.15 (0.89 to 1.47)	0.280
Heart failure	1.15 (0.70 to 1.88)	0.570	1.10 (0.69 to 1.78)	0.680
Diabetes mellitus	1.38 (1.05 to 1.82)	0.019	1.42 (1.09 to 1.84)	0.008
Chronic kidney disease	0.98 (0.58 to 1.66)	0.940	1.17 (0.72 to 1.89)	0.520
Chronic obstructive pulmonary disease	1.53 (1.05 to 2.22)	0.028	1.51 (1.06 to 2.16)	0.023
Active hematological neoplasia	1.85 (0.80 to 4.27)	0.150	1.65 (0.76 to 3.59)	0.210
Active solid tumor	1.59 (0.84 to 2.99)	0.150	1.20 (0.64 to 2.24)	0.570
Use of angiotensin-converting enzyme inhibitor	1.00 (0.73 to 1.36)	1.000	0.83 (0.61 to 1.12)	0.220
Use of angiotensin II receptor blocker	0.91 (0.64 to 1.31)	0.620	0.89 (0.63 to 1.25)	0.490
**Organ support on day 0***				
Use of vasopressor or inotropes	1.11 (0.81 to 1.51)	0.510	1.09 (0.81 to 1.46)	0.570
Fluid balance to mL	1.07 (0.96 to 1.21)	0.230	1.04 (0.93 to 1.16)	0.460
**Oxygenation variables on day 0***				
PaO_2/FiO_2	0.88 (0.76 to 1.01)	0.065	0.88 (0.77 to 1.00)	0.044
**Laboratory tests on day 0***				
Creatinine to µmol/L	1.00 (0.91 to 1.09)	0.980	1.02 (0.94 to 1.10)	0.620
pH	0.71 (0.62 to 0.82)	<0.001	0.73 (0.64 to 0.83)	<0.001
**Vital signs on day 0***				
Mean arterial pressure to mm Hg	0.89 (0.79 to 1.01)	0.066	0.89 (0.79 to 1.00)	0.051
Heart rate to beats per minute	1.07 (0.94 to 1.22)	0.300	1.08 (0.96 to 1.22)	0.210

The models are mixed-effects models with centers as a random effect. *Median value on the first day of invasive ventilation.

Supplementary Table 4. Multivariable assessment of factors associated with hospital and ICU mortality.

	Hospital mortality		ICU mortality	
	**Odds ratio (95% CI)**	**P value**	**Odds ratio (95% CI)**	**P value**
**Age category**				
Age 22 to 57 years	1 (reference)		1 (reference)	
Age 58 to 65 years	1.67 (1.05 to 2.65)	0.030	1.63 (1.03 to 2.58)	0.037
Age 66 to 72 years	3.30 (2.08 to 5.24)	<0.001	3.04 (1.92 to 4.79)	<0.001
Age 73 to 85 years	5.35 (3.33 to 8.61)	<0.001	4.64 (2.90 to 7.42)	<0.001
**Demographic characteristics**				
Male gender	1.48 (1.04 to 2.09)	0.028	1.40 (1.00 to 1.96)	0.051
Body-mass index to kg/m^2	0.99 (0.85 to 1.14)	0.872	0.99 (0.85 to 1.14)	0.845
Hypertension	1.08 (0.75 to 1.54)	0.688	1.00 (0.70 to 1.41)	0.992
Heart failure	0.97 (0.48 to 1.94)	0.923	0.99 (0.50 to 1.95)	0.971
Diabetes mellitus	1.43 (1.00 to 2.05)	0.053	1.44 (1.01 to 2.06)	0.043
Chronic kidney disease	1.42 (0.67 to 3.00)	0.357	1.45 (0.70 to 2.99)	0.321
Chronic obstructive pulmonary disease	1.56 (0.91 to 2.67)	0.108	1.50 (0.89 to 2.51)	0.218
Active hematological neoplasia	2.29 (0.74 to 7.14)	0.152	2.55 (0.85 to 7.66)	0.095
Active solid tumor	1.05 (0.44 to 2.52)	0.916	1.18 (0.50 to 2.81)	0.701
Use of angiotensin-converting enzyme inhibitor	0.78 (0.51 to 1.19)	0.253	0.88 (0.58 to 1.34)	0.556
Use of angiotensin II receptor blocker	0.88 (0.53 to 1.45)	0.600	0.95 (0.58 to 1.55)	0.823
**Organ support on day 0***				
Use of vasopressor or inotropes	1.15 (0.79 to 1.69)	0.465	1.16 (0.80 to 1.69)	0.435
Fluid balance to mL	1.00 (0.86 to 1.17)	0.954	1.05 (0.90 to 1.22)	0.525
**Oxygenation variables on day 0***				
PaO_2/FiO_2	0.86 (0.73 to 1.03)	0.098	0.83 (0.70 to 0.98)	0.031
**Laboratory tests on day 0***				
Creatinine to µmol/L	1.09 (0.92 to 1.29)	0.321	1.07 (0.92 to 1.24)	0.405
pH	0.68 (0.57 to 0.81)	<0.001	0.69 (0.59 to 0.82)	<0.001
**Vital signs on day 0***				
Mean arterial pressure to mm Hg	0.86 (0.73 to 1.01)	0.062	0.87 (0.74 to 1.01)	0.069
Heart rate to beats per minute	1.10 (0.94 to 1.30)	0.245	1.07 (0.91 to 1.26)	0.392

The models are mixed-effects models with centers as a random effect. *Median value on the first day of invasive ventilation.
